# The impact of the multidisciplinary tumor board (MDTB) on the management of pancreatic diseases in a tertiary referral center

**DOI:** 10.1016/j.esmoop.2020.100010

**Published:** 2020-12-14

**Authors:** G. Quero, L. Salvatore, C. Fiorillo, C. Bagalà, R. Menghi, B. Maria, C. Cina, V. Laterza, B. Di Stefano, M.G. Maratta, M. Ribelli, F. Galiandro, G.C. Mattiucci, M.G. Brizi, E. Genco, F. D'Aversa, L. Zileri, F. Attili, A. Larghi, V. Perri, F. Inzani, A. Gasbarrini, V. Valentini, G. Costamagna, R. Manfredi, G. Tortora, S. Alfieri

**Affiliations:** 1 Digestive Surgery Unit, Fondazione Policlinico Universitario Agostino Gemelli, IRCCS, Rome, Italy; 2 CRMPG (Advanced Pancreatic Research Center), Rome, Italy; 3 Università Cattolica del Sacro Cuore di Roma, Rome, Italy; 4 Comprehensive Cancer Center, Fondazione Policlinico Universitario Agostino Gemelli, IRCCS, Rome, Italy; 5 Radiation Oncology Unit, Fondazione Policlinico Universitario Agostino Gemelli, IRCCS, Rome, Italy; 6 Radiology Unit, Fondazione Policlinico Universitario Agostino Gemelli, IRCCS, Rome, Italy; 7 Internal Medicine, Gastroenterology and Hepatology Unit, Fondazione Policlinico Universitario Agostino Gemelli, IRCCS, Rome, Italy; 8 Digestive Endoscopy Unit, Fondazione Policlinico Universitario Agostino Gemelli, IRCCS, Rome, Italy; 9 CERTT, Center for Endoscopic Research Therapeutics and Training, Università Cattolica del Sacro Cuore di Roma, Rome, Italy; 10 Pathology Unit, Università Cattolica del Sacro Cuore di Roma, Rome, Italy

**Keywords:** multidisciplinary tumor board, pancreatic cancer, pancreatic disease management, resectability assessment, tumor response

## Abstract

**Background:**

The implementation of multidisciplinary tumor board (MDTB) meetings significantly ameliorated the management of oncological diseases. However, few evidences are currently present on their impact on pancreatic cancer (PC) management. The aim of this study was to evaluate the impact of the MDTB on PC diagnosis, resectability and tumor response to oncological treatment compared with indications before discussion.

**Patients and methods:**

All patients with a suspected or proven diagnosis of PC presented at the MDTB from 2017 to 2019 were included in the study. Changes of diagnosis, resectability and tumor response to oncological/radiation treatment between pre- and post-MDTB discussion were analyzed.

**Results:**

A total of 438 cases were included in the study: 249 (56.8%) were presented as new diagnoses, 148 (33.8%) for resectability assessment and 41 (9.4%) for tumor response evaluation to oncological treatment. MDTB discussion led to a change in diagnosis in 54/249 cases (21.7%), with a consequent treatment strategy variation in 36 cases (14.5%). Change in resectability was documented in 44/148 cases (29.7%), with the highest discrepancy for borderline lesions. The treatment strategy was thus modified in 27 patients (18.2%). The MDTB brought a modification in the tumor response assessment in 6/41 cases (14.6%), with a consequent protocol modification in four (9.8%) cases.

**Conclusions:**

MDTB discussion significantly impacts on PC management, especially in high-volume centers, with consistent variations in terms of diagnosis, resectability and tumor response assessment compared with indications before discussion.

## Highlights

• Few evidences are present on the impact of the multidisciplinary tumor board (MDTB) on pancreatic cancer (PC) management
• We documented a discrepancy rate of 23.7% between the pre- and post-MDTB discussion.
• This underlines the fundamental role of the MDTB in PC management.

## Introduction

Despite the recent introduction of multimodal treatment strategies, pancreatic cancer (PC) still remains the fourth cause of cancer-related death, and it is expected to become the second by 2030.^1^^,^^2^ Currently, surgical resection represents the mainstay of cure. However, only 15%-20% of patients present a resectable disease, whereas 30%-40% of PCs are defined as locally advanced at first diagnosis, and the remaining 40% present as metastatic disease.^3^ In addition to the low rate of resectability, surgical resection is notably burdened by a significant incidence of related morbidity and mortality.^4^

The recommended centralization of PC treatment in high-volume centers has brought significantly better clinical and surgical outcomes,^5^ thanks to the multidisciplinary approach to the disease and to the consequent dedicated diagnostic and therapeutic pathways. For instance, the multidisciplinary coordination of care is currently recommended in multiple national and international guidelines, here including the National Comprehensive Cancer Network (NCCN).^6^ This multidisciplinary coordination usually takes place in the form of multidisciplinary tumor boards (MDTBs) with the aim of guarantying appropriate disease care, improving outcomes in cancer treatment, standardizing treatment strategies and ensuring an appropriate use of health care sources. In order to accomplish these purposes, MDTBs involve radiologists, surgeons, gastroenterologists, medical and radiation oncologists, endoscopists and pathologists in the decision making. Benefits of MDTBs have already been reported for several oncological diseases, including breast cancer, gastrointestinal, thoracic, urologic and gynecologic malignancies, as well as in the surgical treatment of colorectal liver metastases.^7^, ^8^, ^9^, ^10^, ^11^, ^12^

Conversely, few evidences are currently present on the impact of MDTBs on PC treatment. Given the complexity of PC management, MDTBs could potentially contribute to the appropriate decision making, especially for borderline and locally advanced tumors. These represent 25% of all the PCs and an accurate evaluation is fundamental for an adequate patient selection for surgery, as well as in the timing decision for neoadjuvant/adjuvant treatment. Despite these premises and previous experiences^13^^,^^14^ showing treatment strategy changes after MDBTs in up to 25% of patients affected by PC, the role of the multidisciplinary approach on PC treatment is still largely unknown. The purpose of this study is, thus, to evaluate the impact of MDTBs in a tertiary referral center for PC treatment in terms of clinical and surgical recommendations compared with indications received before the multidisciplinary discussion.

## Methods

### Study population

After Institution Review Board approval, all patients with a proven or suspected diagnosis of PC referred to the MDTBs of the Fondazione Policlinico Universitario Agostino Gemelli IRCCS of Rome from November 2017 to December 2019 were retrospectively included in the study. Biliary tract, duodenal and ampullary tumors, and neuroendocrine neoplasms were excluded from the analysis. Conversely, cases of pancreatitis with suspected underlying tumors were included in the study.

Patients' demographic and clinical data recorded included: age, sex, medical history, symptoms and signs related to the pancreatic disease, laboratory test results and histopathological evaluations (when carried out), at the time of referral. All radiological exams, including computed tomography and/or magnetic resonance imaging and/or positron emission tomography and/or endoscopic ultrasonography were electronically recorded before discussion.

### PC-MDTB

The PC-MDTB was held once a week with the participation of surgeons, gastroenterologists, clinical and radiation oncologists, radiologists, endoscopists and pathologists. All medical records and radiological images were routinely *de novo* reviewed case-by-case during the MDTB and a consensus recommendation was given on the basis of a collective judgment. In case of insufficient diagnostic data, additional exams including radiological exams and/or endoscopic procedures with or without biopsy were prescribed and cases were then re-evaluated.

Indication for discussion was at the discretion of the attending physician. This led to the inclusion of patients at different stages of the diagnostic-therapeutic pathway, such as at diagnosis, before surgery (for resectability assessment) and during/at the end of oncological and/or radiation therapy.

Recommendations on tumor resectability were based on experts' opinion at the time of the discussion and in accordance with the current treatment guidelines, including the US NCCN guidelines.^15^ More specifically, PCs were categorized as resectable, borderline resectable, locally advanced and metastatic.

Indication for and proposal of neoadjuvant, adjuvant or palliative treatments were based on tumor staging, patients' performance status, age and patients' comorbidities, according to the ESMO^16^ and the Italian Medical Oncology Association guidelines.^17^

With regards to intraductal papillary mucinous neoplasms (IPMNs), follow-up or indication for surgical resection was based on the current International Consensus Guidelines 2016.^18^

In case of discrepancies among the members of the MDTB, decisions were taken on the basis of the abovementioned guidelines.

In all cases, pre- and post-MDTB diagnosis, staging and indications were prospectively collected at the time of discussion. Pre-MDTB information was defined as the diagnosis/staging/indications with which the case was presented at the discussion. Post-MDTB information was defined as the collaborative decision, in terms of diagnosis/staging/indications, after the discussion. All concordances or discrepancies between pre- and post-MDTB diagnosis/staging/indications were recorded.

### Study outcomes

The primary outcome of the study was the evaluation of changes in the PC management after MDTB discussion. More specifically, the following three pre- and post-MDTB major features were evaluated and compared (i) tumor diagnosis, (ii) tumor resectability, and (iii) tumor response to oncological and/or radiation treatment.

The allocation of cases to one of the three outcomes groups was based on the first request for discussion by the attending physician (diagnosis, resectability assessment or tumor response to treatment), and each case was allocated to one of the three outcomes groups.

### Statistical analysis

Categorical variables are presented as numbers and percentages, and continuous variables are presented as median and range (min-max). All data were analyzed by SPSS v25® (IBM, Chicago, IL).

## Results

The study population included 378 patients for a total of 438 consecutive cases discussed at the PC-MDTB of the Fondazione Policlinico Universitario Agostino Gemelli IRCCS of Rome from November 2017 to December 2019. The median age at the time of presentation was 65 (15-89) years with 209 (55.3%) males and 169 females (44.7%). Pancreatic adenocarcinomas (242, 55.2%) represented the most frequently discussed diseases, followed by pancreatitis (82, 18.7%), cystic lesions (73, 16.6%) and IPMNs (41, 9.4%). During the study period, 49 (13%) patients were presented more than once. More specifically, 40 cases were discussed twice, seven cases three times and two patients four times. The most common causes of re-discussion were the need of additional examinations at the first evaluation (37 patients, 92.5%) and re-evaluation during chemotherapy (12 patients, 7.5%). Clinical-demographic characteristics of the study cohort are reported in Table 1.

**Table 1:** Clinical-demographic characteristics of the study population

	No. of patients (*n* = 378)
Discussed cases, *n*	438
Age (years), median (range)	65 (15-89)
Sex ratio (M : F)	1 : 1.2
Pancreatic disease (pre-MDTB)	
Adenocarcinoma, *n* (%)	242 (55.2)
Pancreatitis, *n* (%)	82 (18.7)
Cystic lesions, *n* (%)	73 (16.6)
IPMN, *n* (%)	41 (9.4)
Case presentation	
Surgeons, *n* (%)	168 (38.4)
Medical oncologists, *n* (%)	128 (29.2)
Gastroenterologists, *n* (%)	77 (17.6)
Radiation oncologists, *n* (%)	44 (10)
Endoscopists, *n* (%)	21 (4.8)

IPMN, intraductal papillary mucinous neoplasm; MDTB, multidisciplinary tumor board.

The majority of cases (249/438, 56.8%) were discussed as new diagnoses, while 148 (33.8%) patients were assessed for resectability and the remaining 41 (9.4%) cases were discussed for response evaluation to oncological and/or radiation treatment. Of note, PC-MDTB brought a change, as a whole, in 104/438 cases (23.7%).

**Figure 1:**
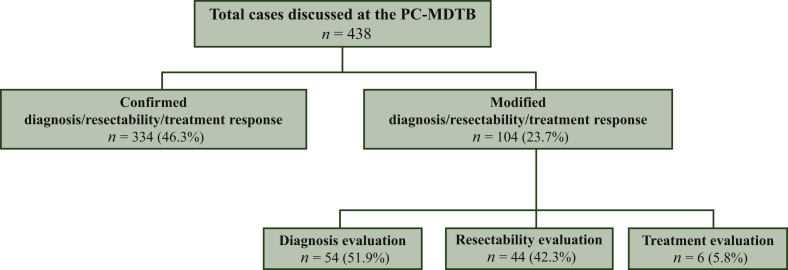
Descriptive flowchart of the study population. PC-MDTB, pancreatic cancer multidisciplinary tumor board.

A descriptive flowchart of the study population is shown in Figure 1.

### PC diagnosis evaluation

**Figure 2:**
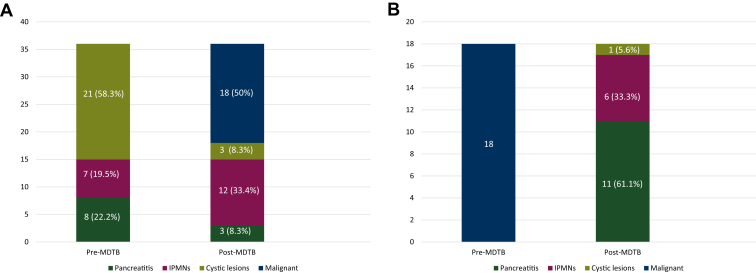
Diagnosis change after MDTB for benign lesions (A) and malignant lesions (B). IPMN, intraductal papillary mucinous neoplasm; MDTB, multidisciplinary tumor board.

Of the 249 cases presented as new diagnoses, 82 (32.9%) were presented as pancreatitis, 73 (29.3%) as cystic lesions, 53 (21.3%) as pancreatic malignant lesions, and 41 (16.5%) as IPMNs. Presentation at the MDTB led to a change of diagnosis in 54 cases (21.7%). More specifically, 36/54 cases (66.7%) firstly presented as benign diseases (pancreatitis, IPMNs or cystic lesions) were defined as pancreatic tumor lesions in 18 cases (50%) after radiological imaging revision and subsequently confirmed at histological analysis. Similarly, 18/54 cases (33.3%) presented as pancreatic malignant lesions before the MDTB resulted in diagnosis of benign diseases after radiological imaging revision. Of these, 11 (61.1%) were diagnosed as focal pancreatitis, six (33.3%) as IPMN and one (5.6%) as a cystic lesion (Figure 2A and B).

For pancreatitis, the discrepancy rate between pre- and post-MDTB was 9.7% (8/82 cases). Of those, three (37.5%) cases were diagnosed as pancreatic adenocarcinoma after MDTB discussion.

Cystic lesions related to a change in diagnosis in 28.8% of cases (21/73): 10/21 cases (47.6%) were pancreatic adenocarcinomas, 9/21 (42.8%) were IPMNs and the remaining two (9.5%) were diagnosed as pseudocysts.

Similarly, a discrepancy rate of 17% (7/41 cases) was evidenced for IPMNs. Of note, 5/7 (71.4%) were diagnosed as pancreatic adenocarcinoma after MDTB discussion.

As a consequence, MDTB discussion brought a treatment plan change in 36 cases (14.5%). Of these, a total of 18 (50%) underwent surgical resection for a new diagnosis of adenocarcinoma and 18 patients (50%), firstly diagnosed with pancreatic malignant lesions, underwent radiological follow-up for a post-MDTB diagnosis of benign disease.

### PC resectability assessment

**Figure 3:**
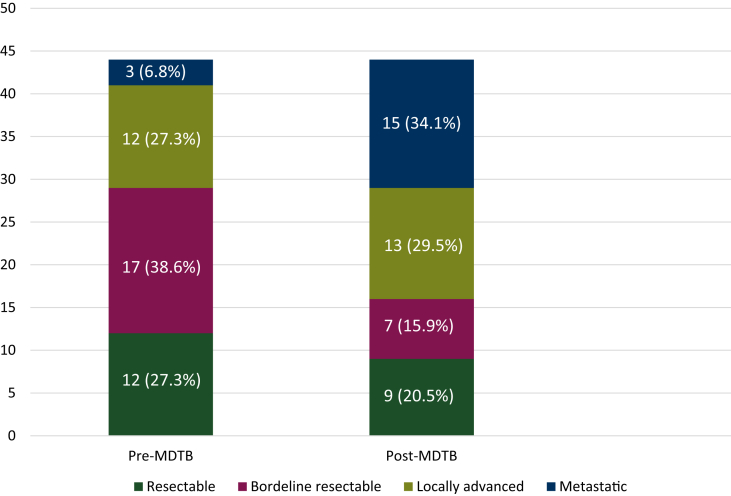
Resectability assessment change after MDTB. MDTB, multidisciplinary tumor board.

A total of 148 cases were discussed for PC resectability assessment. Sixty-two lesions (41.9%) were presented as resectable, 25 (16.9%) as borderline, 44 (29.7%) as locally advanced and 17 (11.5%) as metastatic. MDTB discussion resulted in a change rate of 29.7% (44/148 cases). Resectability assessment change of the 44 cases is reported in Figure 3.

Twelve out 62 cases (19.3%) firstly presented as resectable tumors were classified as locally advanced in three cases (25%), borderline in six cases (50%) and metastatic in three cases (25%) after MDTB discussion.

The highest rate of discrepancy was recorded for cases firstly presented as borderline lesions. In fact, resectability assessment changed in 17/25 patients (68%). Specifically, three (17.7%) had a new diagnosis of resectable disease, 10 (58.8%) of a locally advanced lesion and four (23.5%) of a metastatic tumor.

Of the 44 lesions firstly diagnosed as locally advanced PCs, resectability assessment changed in 12 cases (27.3%). Three patients (25%) had a new diagnosis of resectable disease, one (8.3%) of a borderline tumor and eight (66.7%) of a metastatic disease. For metastatic lesions, the variation rate was 17.6% (three cases). Interestingly, all three cases were classified as resectable tumors after MDTB discussion.

At the final analysis, treatment strategy changed in 27/148 cases (18.2%). Twenty patients (74%), candidates for surgery before MDTB discussion, underwent chemotherapy treatment with neoadjuvant intent and palliative intent in 13 and 7 cases, respectively. Conversely, seven cases (26%), firstly candidates for chemotherapy, underwent surgery as the final agreement of the MDTB discussion.

### Tumor response to oncological and/or radiation treatment

**Figure 4:**
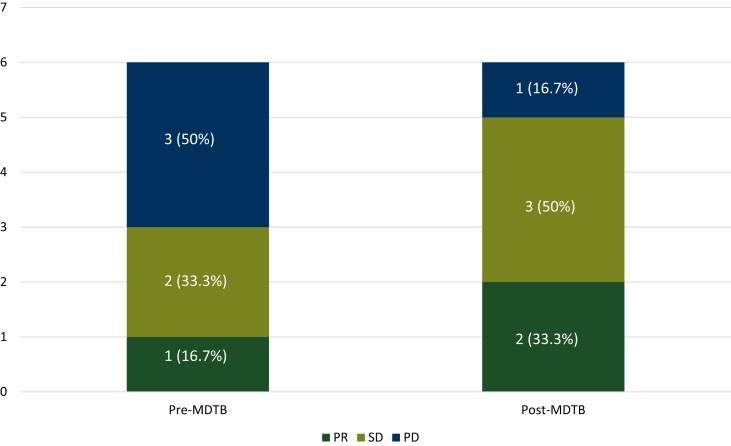
Tumor response assessment change after MDTB. MDTB, multidisciplinary tumor board; PD, progressive disease; PR, partial response; SD, stable disease.

A total of 41 cases were discussed for tumor response evaluation and in six (14.6%) cases, MDTB discussion changed the final disease assessment (Figure 4). Two cases initially presented as stable disease (SD), were classified as partial response (PR) and progressive disease (PD) after MDTB discussion, with a subsequent change of treatment strategy in the last case. Three cases presented as PD after radiological imaging revisions were evaluated as SD (two cases) and PR (one case), thus allowing the prosecution of an active treatment. Finally, one case classified as PR was changed to SD. Overall, MDTB discussion changed the treatment strategy in 4/41 (9.8%) cases.

## Discussion

The treatment centralization of most oncological diseases and the progressive implementation of MDTBs have demonstrated a relevant impact on patients' outcomes. For instance, the introduction of the multidisciplinary approach has led to a more accurate disease staging, a shorter time lapse between diagnosis and treatment and a lower incidence of post-operative complications for several oncological diseases.^12^^,^^13^^,^^19^^,^^20^

Despite these premises, the role of MDTBs on PC treatment is not yet well established. Therefore, the aim of our retrospective analysis was to investigate the utility and potential benefits of an MDTB for PC in terms of diagnosis, resectability and tumor response after medical treatment compared with the pre-discussion assessment. Of note, we documented a discrepancy rate of 23.7% in the whole population, with the highest discordance rate for tumor resectability assessment (44/148 cases, 29.7%).

In this last regard, an accurate selection of patients for surgery, through an appropriate disease staging, represents a crucial and fundamental task for patients' prognosis.^21^^,^^22^ However, the results published by Bilimoria et al.^23^ give a surprising picture of the surgical treatment of PC in a nationwide setting. The authors reported a significant underuse of pancreatectomy especially for T1 lesions, with a lack of surgical treatment in 71.4% of this population, and with the highest rate of nontreatment registered in low-volume centers. These results underline the central role of specialized institutions in the management of pancreatic diseases, in order to adequately assess diagnosis, staging and resectability, and to guarantee a multidisciplinary and patient-tailored diagnostic and therapeutic pathway.

Currently, the main difficulty in the assessment of PC resectability is represented by the discrimination between borderline and locally advanced lesions. This is mainly due to the presence of multiple and different guidelines and classifications^24^ that lead to the lack of a definitive and uniform consensus on the definition of criteria for PC surgery. In this context, the PC-MDTB plays a key role, thanks to the expertise of dedicated radiologists and to the contemporary risk/benefit ratio evaluation of surgical resection by clinicians and surgeons.

According to our data, the highest discrepancy between pre- and post-MDTB resectability assessment was found for borderline lesions (68%), followed by locally advanced (27.3%), resectable (19.3%) and metastatic tumors (17.6%). The MDTB discussion, thus, led to a treatment strategy change in the 18.2% of cases (27/148). In particular, 20 patients firstly defined as resectable underwent chemotherapy treatment, while seven patients who were candidates for chemotherapy were allocated to surgery after MDTB discussion. Our data do not significantly differ from the current evidences present in the literature. Two previous experiences^13^^,^^14^ documented a change in the treatment allocation in 25% of cases after MDTB evaluation. Moreover, the multidisciplinary imaging revision resulted in a higher percentage of metastatic disease detection in the cohort study presented from the Johns Hopkins experience,^14^ avoiding surgery in a consistent number of cases. Based on these premises, we might speculate potential benefits of the PC-MDTB even in terms of long-term outcomes. Although we did not perform a survival analysis of our cohort, the multidisciplinary approach has already demonstrated an improved prognosis in a recent north European study,^25^ thanks to dedicated specialists and to the management of the disease in referral centers.

With regards to the role of the MDTB in the definition of pancreatic diagnoses, we documented a discrepancy rate of 21.7% (54/249 cases). Interestingly, 36/54 lesions (66.7%) firstly classified as benign were defined as malignant after multidisciplinary discussion. Conversely, 18 (33.3%) pre-MDTB malignant lesions were defined as benign after radiological re-evaluation.

For instance, PC characterization still represents a challenge for radiologists, especially in the case of solid lesions smaller than 2 cm and for those tumors that do not cause deformity of the pancreatic parenchyma.^26^ Of note, our rate of change in diagnosis is significantly higher in comparison to the results reported by Hansen et al.^27^ The authors registered a change rate in only 12.4% of patients. Multiple factors could justify this discrepancy. First, and more importantly, the execution of the majority of baseline clinical and radiological evaluations in low volume and non-dedicated centers, potentially lead to low-quality imaging exams and/or misinterpretation of radiological images by non-dedicated radiologists. Furthermore, the lack of second level exams at the time of the presentation may have caused an initial incorrect diagnosis.

As reported in previous experiences,^14^^,^^27^ imaging revision by radiologists with extensive experience significantly improve the diagnostic accuracy other than the staging assessment. Moreover, the multidisciplinary setting permits appropriate prescription and performance of additional examinations, when needed. In this regard, our institution has dedicated diagnostic and therapeutic pathways. Patients evaluated at the PC-MDTB are contacted the day of the discussion and further examinations, when prescribed, are carried out in a short time lapse (within a week after discussion) at our institution and subsequently re-discussed at the multidisciplinary meeting. This permits significant reduction in the time lapse between diagnosis and patients' allocation to surgical or medical treatment or follow-up.

Regarding the role of the MDTB in tumor response assessment, our analysis showed a discrepancy rate of 14.6% (6/41 cases) with a significant change of the treatment strategy in 9.8% (4/41), thus avoiding continuing ineffective treatments or stopping active ones early. The assessment of tumor response to oncological and/or radiation treatment is insidious and, even in this case, the MDTB plays a crucial role. Radiological imaging revision by dedicated radiologists is fundamental,^28^ as well as setting the patient in a specific clinical context (taking into account further information, such as Ca 19.9 serum levels, general conditions, type of treatment, etc.) thanks to the contemporary involvement of specialized clinicians (medical oncologists, radiation oncologists and gastroenterologists). This approach allows the treatment strategy to be maximized and subsequent improvement in the outcome of patients with PC.

Despite the clear advantages of the PC-MDTB in terms of accuracy for diagnosis, resectability and tumor response to treatment evaluation, our study presents some limitations. The retrospective study design and the monocentric origin of the data represent one of the main drawbacks. In addition, the lack of a comparative group of no-MDTB patients does not permit drawing conclusions in terms of time-lapse between diagnosis and treatment, and short-term outcomes. Similarly, the absence of a long-term follow-up analysis does not allow evaluation of the potential benefits of the PC-MDTB on patients' prognosis.

In conclusion, the multidisciplinary approach to PC in high-volume centers is fundamental in all steps of the disease management. The dramatic change in the treatment strategy we registered further confirms the PC-MDTB as efficient, effective and essential from diagnosis to the resectability assessment and tumor response evaluation after medical treatment. However, additional studies, including a comparative cohort of no-MDTB patients, are needed to support our results.

## References

[1] Rahib L., Smith B.D., Aizenberg R., Rosenzweig A.B., Fleshman J.M., Matrisian L.M. (2014). Projecting cancer incidence and deaths to 2030: the unexpected burden of thyroid, liver, and pancreas cancers in the United States. Cancer Res.

[2] Siegel R.L., Miller K.D., Jemal A. (2019). Cancer statistics, 2019. CA Cancer J Clin.

[3] Ryan D.P., Hong T.S., Bardeesy N. (2014). Pancreatic adenocarcinoma. N Engl J Med.

[4] Kimura W., Miyata H., Gotoh M. (2014). A pancreaticoduodenectomy risk model derived from 8575 cases from a national single-race population (Japanese) using a web-based data entry system: the 30-day and in-hospital mortality rates for pancreaticoduodenectomy. Ann Surg.

[5] Lidsky M.E., Sun Z., Nussbaum D.P., Adam M.A., Speicher P.J., Blazer D.G. (2017). Going the extra mile: improved survival for pancreatic cancer patients traveling to high-volume centers. Ann Surg.

[6] Tempero M.A., Malafa M.P., Behrman S.W. (2014). Pancreatic adenocarcinoma, version 2.2014. J Natl Compr Canc Netw.

[7] Chang J.H., Vines E., Bertsch H. (2001). The impact of a multidisciplinary breast cancer center on recommendations for patient management: the University of Pennsylvania experience. Cancer.

[8] Greer H.O., Frederick P.J., Falls N.M. (2010). Impact of a weekly multidisciplinary tumor board conference on the management of women with gynecologic malignancies. Int J Gynecol Cancer.

[9] Lamb B.W., Green J.S., Benn J., Brown K.F., Vincent C.A., Sevdalis N. (2013). Improving decision making in multidisciplinary tumor boards: prospective longitudinal evaluation of a multicomponent intervention for 1,421 patients. J Am Coll Surg.

[10] Modest D.P., Denecke T., Pratschke J. (2018). Surgical treatment options following chemotherapy plus cetuximab or bevacizumab in metastatic colorectal cancer-central evaluation of FIRE-3. Eur J Cancer.

[11] Santoso J.T., Schwertner B., Coleman R.L., Hannigan E.V. (2004). Tumor board in gynecologic oncology. Int J Gynecol Cancer.

[12] van Hagen P., Spaander M.C., van der Gaast A. (2013). Impact of a multidisciplinary tumour board meeting for upper-GI malignancies on clinical decision making: a prospective cohort study. Int J Clin Oncol.

[13] Brauer D.G., Strand M.S., Sanford D.E. (2017). Utility of a multidisciplinary tumor board in the management of pancreatic and upper gastrointestinal diseases: an observational study. HPB (Oxford).

[14] Pawlik T.M., Laheru D., Hruban R.H. (2008). Evaluating the impact of a single-day multidisciplinary clinic on the management of pancreatic cancer. Ann Surg Oncol.

[15] Tempero M.A., Malafa M.P., Chiorean E.G. (2019). NCCN guidelines insights: pancreatic adenocarcinoma, version 1.2019: featured updates to the NCCN guidelines. J Natl Compr Canc Netw.

[16] Ducreux M., Caramella C., Hollebecque A. (2015). Cancer of the pancreas: ESMO Clinical Practice Guidelines for diagnosis, treatment and follow-up. Ann Oncol.

[17] Silvestris N., Brunetti O., Bittoni A. (2020). Clinical practice guidelines for diagnosis, treatment and follow-up of exocrine pancreatic ductal adenocarcinoma: evidence evaluation and recommendations by the Italian Association of Medical Oncology (AIOM). Cancers.

[18] Tanaka M., Fernández-del Castillo C., Kamisawa T. (2017). Revisions of international consensus Fukuoka guidelines for the management of IPMN of the pancreas. Pancreatology.

[19] Basta Y.L., Baur O.L., van Dieren S., Klinkenbijl J.H., Fockens P., Tytgat K.M. (2016). Is there a benefit of multidisciplinary cancer team meetings for patients with gastrointestinal malignancies?. Ann Surg Oncol.

[20] Pillay B., Wootten A.C., Crowe H. (2016). The impact of multidisciplinary team meetings on patient assessment, management and outcomes in oncology settings: a systematic review of the literature. Cancer Treat Rev.

[21] Conroy T., Desseigne F., Ychou M. (2011). FOLFIRINOX versus gemcitabine for metastatic pancreatic cancer. N Engl J Med.

[22] Neoptolemos J.P., Palmer D.H., Ghaneh P. (2017). Comparison of adjuvant gemcitabine and capecitabine with gemcitabine monotherapy in patients with resected pancreatic cancer (ESPAC-4): a multicentre, open-label, randomised, phase 3 trial. Lancet.

[23] Bilimoria K.Y., Bentrem D.J., Ko C.Y., Stewart A.K., Winchester D.P., Talamonti M.S. (2007). National failure to operate on early stage pancreatic cancer. Ann Surg.

[24] Kumar R., Herman J.M., Wolfgang C.L., Zheng L. (2013). Multidisciplinary management of pancreatic cancer. Surg Oncol Clin N Am.

[25] Kersten C., Cvancarova M., Mjaland S., Mjaland O. (2013). Does in-house availability of multidisciplinary teams increase survival in upper gastrointestinal-cancer?. World J Gastrointest Oncol.

[26] Feldman M.K., Gandhi N.S. (2016). Imaging evaluation of pancreatic cancer. Surg Clin North Am.

[27] Hansen M.F.C., Storkholm J.H., Hansen C.P. (2020). The results of pancreatic operations after the implementation of multidisciplinary team conference (MDT): a quality improvement study. Int J Surg.

[28] Baliyan V., Kordbacheh H., Parakh A., Kambadakone A. (2018). Response assessment in pancreatic ductal adenocarcinoma: role of imaging. Abdom Radiol (NY).

